# Common Genetic Variants in Rare Disorders: Hematology and Beyond

**DOI:** 10.3390/cimb47010023

**Published:** 2025-01-01

**Authors:** Paschalis Evangelidis, Maria Gavriilaki, Nikolaos Kotsiou, Eleni Gavriilaki

**Affiliations:** 1 Second Propedeutic Department of Internal Medicine, Aristotle University of Thessaloniki, 54642 Thessaloniki, Greece; pascevan@auth.gr (P.E.); kotsiounikolao@gmail.com (N.K.); 2 1st Department of Neurology, AHEPA University Hospital, Aristotle University of Thessaloniki, 54636 Thessaloniki, Greece; mariagavri6@yahoo.gr; 3 Hematology Department and Bone Marrow Transplant (BMT) Unit, G. Papanicolaou Hospital, 57010 Thessaloniki, Greece

Emerging evidence suggests that common genetic variants play a significant role in various rare but life-threatening hematological and non-hematological conditions. Transplant-associated thrombotic microangiopathy (TA-TMA) constitutes a complication of hematopoietic cell transplantation (HCT), which is considered the only curative option for many malignant and benign hematological disorders. TA-TMA is characterized by microangiopathic hemolytic anemia, thrombocytopenia, and organ damage, such as kidney failure and central nervous system involvement [1]. The pathophysiology of this syndrome is based on endothelial injury and activation of the complement system [2]. Based on this hypothesis, our group investigated the role of genetic susceptibility in the development of TA-TMA using next-generation sequencing (NGS) in 40 allogeneic HCT (allo-HCT) recipients, 18 HCT donors, and 40 healthy controls [3]. The frequency of common genetic variants related to complement activation and endothelial dysfunction was significantly increased in TA-TMA patients compared to controls and HCT donors. The identified variants were also described in other rare clinical entities, such as atypical hemolytic uremic syndrome (aHUS), C3G nephropathy, and age-related macular degeneration, which share a common pathogenic mechanism: increased complement activation. Variants in genes related to endothelial function and folate metabolism have been described as possible risk factors for the development of sinusoidal obstruction syndrome/veno-occlusive disease (SOS/VOD), a rare but severe allo-HCT complication [4,5]. More data regarding the clinical significance of genetic susceptibility to SOS/VOD post-allo-HCT are crucial.

Beyond hematological disorders, endothelial injury is implicated in the pathogenesis of several other diseases. Various in vitro experiments have shown that endothelial cell activation plays a crucial role in the development of cardiovascular disease [6]. Moreover, a crosstalk between endothelium and hematopoietic stem cells is well described [7,8]. Based on this hypothesis, our group showed that allo-HCT recipients experience an increased burden of cardiovascular risk in comparison to healthy individuals of the same age [9,10].

In addition to endothelial injury syndromes, observed in HCT settings, TMAs might cause complications in various other clinical conditions. Catastrophic antiphospholipid syndrome (APS) is an ultra-rare complication of APS, characterized by multiple organ failure. Mutations in complement regulatory genes have been identified in APS patients [11]. Moreover, similar mutations were recognized by our group in patients who developed drug-induced TMA, such as those with multiple myeloma treated with carfilzomib, an irreversible proteasome inhibitor [12].

In the era of precision medicine, artificial intelligence networks incorporating genetic data have been effectively used to predict outcomes in patients with non-hematological disorders, such as in our work on COVID-19 [13]. Similarly to TMAs, variants in complement and endothelium-related genes have been correlated with severe COVID-19 [14,15]. The pathogenesis of severe COVID-19 syndrome is similar to that of various TMAs, while complementary activation is also implicated [16,17,18].

In patients with b-thalassemia and sickle cell disease, in addition to the well-known genetic background implicated in their pathogenesis, common SNPs have been recognized to play a significant role in the development of disease-related complications, such as diabetes mellitus, osteoporosis, and sickle cell crisis [19,20]. Additionally, heme, as a component of hemoglobin, interacts with various molecules, including complement system factors, leading to vicious cycle of hemolysis and inflammation [21,22]. The International Hemoglobinopathy Research Network (INHERENT) and HELIOS cost action are working in this field to collect genomic data from patients living with hemoglobinopathies to better understand the association of genetic variants and SNPs with specific phenotypes and complications. Multicenter collaboration is essential to achieve this aim [23].

There is great interest in understanding the molecular background of various diseases and published studies mainly focus on predisposition through SNPs. This is of paramount importance, but in many cases, the molecular mechanism implicated in the disease’s pathogenesis can be more complex, because in various cases, what makes the difference is the combination of major and minor alleles in multiple haplotypes. In the era of personalized medicine, we need more data regarding the clinical significance of these data and interdisciplinary collaboration can play a key role in this interdisciplinary field.
